# Calcium silicate enhances immunosuppressive function of MSCs to indirectly modulate the polarization of macrophages

**DOI:** 10.1093/rb/rbab056

**Published:** 2021-10-22

**Authors:** Haiyan Li, Wenrui Wang, Jiang Chang

**Affiliations:** 1 School of Biomedical Engineering, Shanghai Jiao Tong University, 1954 Huashan Road, Shanghai 200030, China; 2 Chemical and Environment Engineering Department, School of Engineering, RMIT University, 124 La Trobe Street, Melbourne, VIC 3001, Australia; 3 State Key Laboratory of Performance Ceramics and Superfine Microstructure, Shanghai Institute of Ceramics, Chinese Academy of Sciences, 1295 Dingxi Road, Shanghai 200050, China

**Keywords:** calcium silicate, MSCs, macrophage, immunomodulation

## Abstract

Bioactive silicate ceramics (BSCs) have been widely reported to be able to induce bone tissue regeneration, but the underlying mechanisms have not been fully elucidated. Previous studies have reported that ionic products of BSCs can promote bone regeneration by directly simulating osteogenic differentiation of mesenchymal stem cells (MSCs) and modulating the polarization of macrophages to create a favorable inflammation microenvironment for initiating bone regeneration cascades. However, the immunomodulatory ability of MSCs also plays a critical role in bone regeneration but the effects of BSCs on the immunomodulatory ability of MSCs have been rarely investigated. This study aims to investigate the effects of ionic products of BSCs on the immunoregulatory ability of MSCs to further understand the mechanism of BSCs enhancing bone regeneration. Results showed that ionic products of calcium silicate (CS), one of the representative BSCs, could enhance the immunosuppressive function of human bone marrow mesenchymal stem cells (HBMSCs) by up-regulating the expression of immunosuppressive factors in HBMSCs via NF-κB pathway. In addition, CS-activated HBMSCs showed stronger stimulatory effects on M2 polarization of macrophages than CS ionic products. Furthermore, the macrophages educated by CS-activated HBMSCs showed stronger stimulatory effects on the early osteogenic differentiation of HBMSCs than the ones regulated by CS ionic products. These results not only provide further understanding on the mechanism of BSCs enhancing bone regeneration but also suggest that it is critical to consider the effects of biomaterials on the immunomodulatory function of the tissue forming cells when the immunomodulatory function of biomaterials is investigated.

## Introduction

During the past few decades, bioactive silicate ceramics (BSCs) have been widely reported to have great application potential for bone repair due to their good osteoinductivity. For advancing the development of biomedical BSC products, the mechanisms of BSCs on inducing bone regeneration have been intensively studied. At first, it was discovered that BSCs could induce the formation of a carbonate substituted hydroxyapatite layer on their surface when exposed to body fluid, which allowed the materials to chemically bond to living bone and thus promoted the formation of new bone [1–3]. Then, with the increasing understanding of the interactions between biomaterials and living cells, more and more studies have demonstrated that the ionic dissolution products (e.g. Si, Ca, P) from BSCs are able to stimulate specific cellular responses associated with the process of osteogenesis and angiogenesis, such as promoting the and differentiation of osteoblasts [4–6] and mesenchymal stem cells (MSCs) [7–9], activating the secretion of growth factors from fibroblasts [10] and endothelial cells [11, 12] and regulating the interaction between endothelial cells and stem cells or fibroblasts [13–15]. However, the mechanisms of the biological function of BSCs are still unknown, which is crucial for design of bioactive bone repairing materials. Some previous studies have shown that osteoimmunomodulatory property of bone biomaterials may play a key role [16], and evidences have emerged that many BSCs can promote the bone regeneration through directly modulating the inflammation responses of host by regulating the polarization of macrophages [17–20]. For example, Chen *et al*. [17] reported that Sr, Zn and Si ions released from Sr_2_ZnSi_2_O_7_ could inhibit the secretion of pro-inflammatory cytokines and fibrosis-enhancing factors of macrophages, and the conditioned medium from macrophages stimulated with Sr_2_ZnSi_2_O_7_ could significantly enhance the osteogenic differentiation of bone marrow MSCs (BMSCs). Wu *et al*. [18] reported that clinoenstatite (MgSiO_3_) could enhance osteogenesis and inhibit osteoclastogenesis by regulating macrophages to polarize into M2 phenotype. However, previous studies mainly focus on the direct effects of BSCs on the behaviors of macrophages without considering the immunomodulatory effects of other cells on macrophages.

In the process of bone regeneration, it is well known that MSCs can be recruited from bone marrow, periosteum and blood circulation to the injury site under the induction of inflammatory factors and chemokines released by macrophages to further proliferate, differentiate and secrete extracellular matrix for forming bone tissue [21–23]. Recently, more and more studies have reported that MSCs can affect the immune microenvironment and subsequently adjust the functions of various innate immune cells and adaptive immune cells, so that a balanced inflammation and regenerative microenvironment can form for promoting the bone regeneration and repair [24]. Many studies have confirmed that the mechanism by which MSCs regulate macrophage phenotype polarization is closely related to its paracrine function [25–30]: in response to the stimulation of inflammatory factors produced by M1 type macrophages, MSCs first recruit macrophages to themselves by releasing chemokines. Under TNF-α stimulation, MSCs secrete tumor necrosis factor-inducible gene 6 protein (TSG-6) and act on the recruited macrophages, thereby inhibiting the expression of TNF-α and other pro-inflammatory factors through a negative feedback loop and weakening the pro-inflammatory cascade of macrophages. Meanwhile, MSCs up-regulate the expression of cyclooxygenase 2 (COX-2) and indoleamine 2,3-dioxygenase (IDO) and catalyze the synthesis of metabolites, such as prostaglandin E2 (PGE2) and kynurenic acid (KYNA), thereby stimulating macrophages to express M2 phenotypic markers and secrete IL-10 and transform M1 type macrophages into M2 type macrophages.

Based on the findings on the critical roles of immunomodulation function of MSCs in tissue regeneration, the effects of biomaterials on immunomodulation function of MSCs attract intensive attention. Increasing evidences have shown that the biomaterials can regulate the immunomodulation function of MSCs with structural and mechanical signals, including the surface microstructure [31] and roughness of biomaterial surfaces [32, 33], as well as fiber arrangement [34, 35] etc. For example, Wan *et al*. [34] found that adipose-derived MSCs (Ad-MSCs) cultured on a polylactic acid (PLLA) electrospun membranes with orderly arranged nanofibers could secrete more immunosuppressive molecules TSG-6 and COX-2 than those cultured on a PLLA electrospun membranes with randomly arranged nanofibers. More interestingly, the conditioned medium of Ad-MSCs cultured on electrospun membranes with orderly arranged nanofibers can promote the polarization of macrophages toward the M2 phenotype. However, the effects of chemical signals of biomaterials on immunoregulatory function of MSCs have been rarely reported. More recently, Lima *et al*. [36] reported that high concentration (5 mM) of Mg ions could significantly increase the secretion of immunosuppressive molecule PGE2 from MSCs, and the conditioned medium of MSCs stimulated by Mg ions could inhibit the expression of inflammatory factor TNF-α, IL-1β and IL-6 but promote the expression of anti-inflammatory factor IL-10 in macrophages, suggesting that some biologically active chemical ions may have ability to regulate the immunomodulatory properties of MSCs.

Since the ionic products of BSCs have been confirmed to have various biological effects on MSCs, such as influencing proliferation and differentiation of MSCs, it can be hypothesized that the ionic products of BSCs may also be able to modulate the immunomodulation function of MSCs and subsequently affect the behaviors of macrophages to adjust the inflammation microenvironment for inducing bone regeneration. To prove this hypothesis for providing further understand on the mechanism of the BSCs in promoting bone regeneration, in this study, calcium silicate (CS) ceramics (CaSiO_3_, CS) was selected as the representative of BSCs. The effects of CS ionic products on the immunosuppressive function of human bone marrow MSCs (HBMSCs) were firstly investigated and the molecular mechanism was studied. Then, the effects of CS ionic products and activated HBMSCs by CS ionic products on the behaviors of macrophages were investigated and compared. After that, the macrophages regulated by CS ionic products and macrophages modulated by CS-activated HBMSCs on the osteogenic differentiation of HBMSCs were evaluated and compared.

## Materials and methods

### Preparation of CS ion products

CS powders were provided by Shanghai Institute of Ceramics, Chinese Academy of Sciences. To prepare CS ionic products (CS ion extracts), 1 g CS powder was soaked in 5 ml of serum-free Mesenchymal Stem Cell Medium (MSCM, ScienCell, USA) according to the method adapted from previous literature and ISO10993-5 [37, 38], and incubated in a humidified 37°C/5% CO_2_ incubator for 24 h. The supernatant was then collected and sterilized through a filter (Millipore, 0.22 μm) and stored at 4°C for further use.

### Cell culture

HBMSCs and human acute monocytic leukemia THP-1 cells were both purchased from Zhong Qiao Xin Zhou Biotechnology Co., Ltd (Shanghai). HBMSCs were cultured with MSCM + 10% fetal bovine serum (FBS, ScienCell) + 1% penicillin/streptomycin (P/S, ScienCell) + 1% Mesenchymal Stem Cell Growth Supplement (MSCGS, ScienCell). Culture medium was refreshed every other day and the cells were passaged at 80% confluence. HBMSCs at passages 3–7 were used.

THP-1 cells were grown in Roswell Park Memorial Institute 1640 (RPMI-1640) complete medium (Zhong Qiao Xin Zhou Biotechnology Co., Ltd., Shanghai) containing 10% FBS, 1% P/S and 0.05 mM β-mercaptoethanol, and the cells were subcultured before reaching the density of 1 × 10^6^ cells/ml. For inducing the differentiation of THP-1 cells into macrophages, 75 ng/ml phorbol 12-myristate 13-acetate (PMA, Sigma, USA) was added to the medium to culture THP-1 cells for 72 h. After that, the obtained macrophages were washed three times with Dulbecco’s Phosphate-Buffered Saline (DPBS, Gibco) prior to be used. All cells were maintained at 37°C in a humidified 5% CO_2_ incubator.

### Effects of CS ionic products on the immunomodulation function of HBMSCs

To investigate the effects of CS ionic products on the immunomodulation function of HBMSCs, HBMSCs were first seeded at the density of 5 × 10^5^ cells per well in six-well plates and cultured with HBMSCs control medium (MSCM + 10% FBS + 1% P/S + 1% MSCGS). Meanwhile, the as prepared CS ion extracts were diluted with the HBMSCs control medium at the ratio of 1/64 and 1/128, which were named as MSCM CS 1/64 and MSCM CS 1/128, respectively. CS ion extracts diluted with the above ratios have been proved to be biocompatible and bioactive to various kinds of cells in our previous studies with proper Si ion concentrations [12, 14, 39]. After HBMSCs were cultured with the control medium for 24 h, culture media of some cells was changed into MSCM CS 1/64 and MSCM CS 1/128 and other cells were continued to be cultured with HBMSCs control medium (MSCM CS 0). All cells were continued to be incubated for 24 h with different media before they were detected in terms of the expression of immunoregulatory factors with immunofluorescence staining assay, western blot analysis and quantitative real-time polymerase chain reaction (Q-RT-PCR), as described in sections ‘Immunofluorescence staining assay’, ‘Western blot analysis’ and ‘Quantitative real-time polymerase chain reaction’.

### Synergistical stimulatory effects of CS ionic products and pro-inflammatory cytokines on the immunomodulation function of HBMSCs

It is known that high levels of pro-inflammatory cytokines interferon-γ (IFN-γ) and tumor necrosis factor-α (TNF-α) can stimulate MSCs to produce immunomodulatory factors. To investigate whether the CS ionic products and pro-inflammatory cytokines (IFN-γ and TNF-α) have synergistic effects on the gene expression of immunoregulatory factors in HBMSCs, HBMSCs were stimulated with CS ionic products, pro-inflammatory cytokines IFN-γ and TNF-α and CS ionic products combined with IFN-γ and TNF-α, respectively. Briefly, HBMSC were first seeded at density of 5 × 10^5^ cells per well in six-well plates and cultured with HBMSCs control medium for 24 h. Then, the cultured medium of HBMSCs was replaced with MSCM CS 0, MSCM CS 1/64 and MSCM CS 1/128 with or without 10 ng/ml IFN-γ (Peprotech, US) and 10 ng/ml TNF-α (Peprotech, US) and the cells were cultured for another 24 h. At the end, the expression of immunoregulatory factors in HBMSCs cultured with different media was detected with Q-RT-PCR, as described in section ‘Quantitative real-time polymerase chain reaction’.

### Mechanism study on the stimulatory effects of CS ionic products on the immunomodulation function of HBMSCs

To further investigate whether the enhancement of the gene expression of immunoregulatory factors in CS-activated HBMSCs is associated to the activation of NF-κB, which has been widely reported to be involved in the production of immunoregulatory factors in response to TNF-α and external stimuli [27, 35, 40, 41], effects of CS ionic products on the gene expression of immunoregulatory factors in HBMSCs pre-treated with or without NF-κB inhibitor were examined. Briefly, HBMSC were first seeded at density of 5 × 10^5^ cells per well in six-well plates and cultured with HBMSCs control medium for 24 h. Then, the culture media of some cells was changed into MSCM CS 1/64, and other cells were continued to be cultured with HBMSCs control medium (MSCM CS 0). For inhibiting NF-κB pathway in HBMSCs, 1 mM NF-κB inhibitor, pyrrolidinedithiocarbamic acid ammonium salt (PDTC, Beyotime, China) was added to half of the MSCM CS 0 and MSCM CS 1/64 groups while another half of groups was kept as original. Then, the gene expression of immunoregulatory factors in HBMSCs pre-treated with or without NF-κB inhibitor were examined with Q-RT-PCR, as described in section ‘Quantitative real-time polymerase chain reaction’.

### Direct and indirect effects of CS ionic products on polarization of macrophages

**Figure 1.**
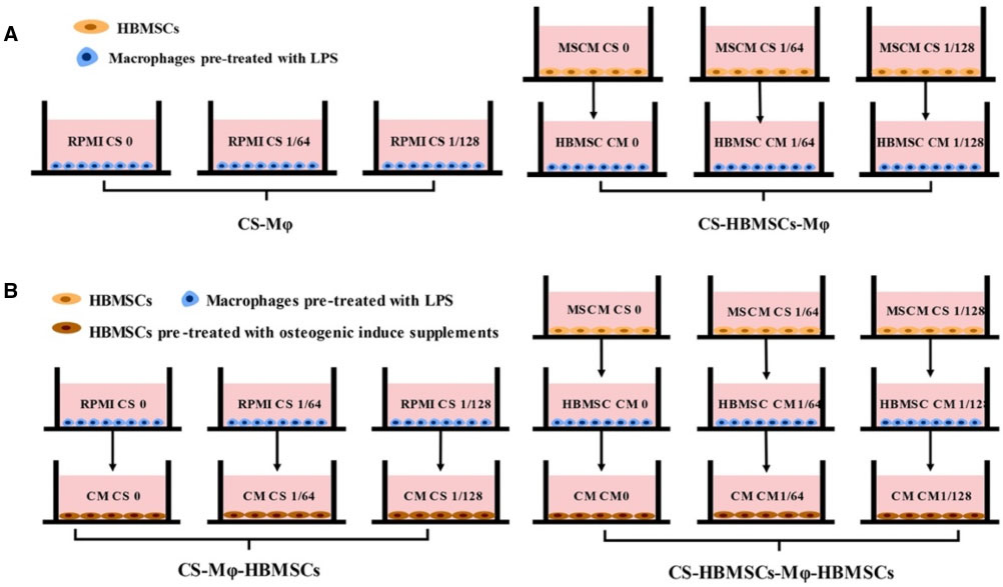
(**A**) Schematic diagram of cell culture methods for evaluating the direct effects of CS ionic products on macrophages and indirect effects of CS-activated HBMSCs on macrophages. The two types of macrophages are referred as CS-Mφ and CS-HBMSCs-Mφ, respectively. (**B**) Schematic diagram of cell culture methods for evaluating the effects of CS-Mφ and CS-HBMSCs-Mφ on the osteogenic differentiation of HBMSCs. The two types of HBMSCs are referred as CS-Mφ-HBMSCs and CS-HBMSCs-Mφ-HBMSCs, respectively

CS ion extracts were diluted with RPMI-1640 complete medium at ratios of 1/64 and 1/128, which were named as RPMI CS 1/64 and RPMI CS 1/128, respectively, while RPMI-1640 complete medium was referred as RPMI CS 0. Then, the direct and indirect effects of CS ionic products on polarization of macrophages were investigated by the protocol illustrated in Fig.  1A. THP-1 cells were firstly seeded in a six-well plate at the density of 1 × 10^6^ cells per well and treated with 500 ng/ml LPS for 1 h to be differentiated into macrophages. Then, for investigating the direct effects of CS ionic products on polarization of macrophages, the macrophages were incubated with RPMI CS 0, RPMI CS 1/64, RPMI CS 1/128 for 48 h. Meanwhile, for investigating the indirect effects of CS ionic products on polarization of macrophages, the macrophages were incubated with conditioned medium collected from CS-activated HBMSC. Briefly, HBMSCs were first seeded at the density of 5 × 10^5^ cells per well in six-well plates and cultured with HBMSCs control medium (MSCM + 10% FBS + 1% P/S + 1% MSCGS) for 24 h before the culture media of some cells was changed into MSCM CS 1/64 and MSCM CS 1/128 and other cells were continued to be cultured with HBMSCs control medium (MSCM CS 0). After 24 h, the culture medium was collected from HBMSCs cultured with MSCM CS 0, MSCM CS 1/64 and MSCM CS 1/128 and referred to HBMSC conditioned medium 0 (HBMSC CM 0), conditioned medium 1/64 (HBMSC CM 1/64) and conditioned medium 1/128 (HBMSC CM 1/128). All conditioned media were filtered with 0.22 μm filter (Millipore) before they were diluted with RPMI-1640 complete medium at a ratio of 1:1 and the diluted media were used to culture the pre-treated macrophages for 48 h. The macrophages regulated by CS ionic products and conditioned medium of CS-activated HBMSCs were referred as CS-Mφ and CS-HBMSCs-Mφ (Fig.  1A), respectively. The polarization of the macrophages was evaluated by Q-RT-PCR and flow cytometry, as described in section ‘Quantitative real-time polymerase chain reaction’ and as following, respectively.

For flow cytometry assay, after the THP-1 cells were incubated with different media, the cells were gently scraped off and washed three times with DPBS and blocked with 1% bovine serum albumin (BSA)–DPBS for 30 min at 37°C. Then, the cells were washed and incubated with CCR7 (1:20, eBioscience, USA) or CD163 (1:20, eBioscience) at 4°C for 30 min. Finally, the stained cells were resuspended in 600 μl DPBS after being washed two times and analyzed by using a flow cytometer (FACS Aria II, BD).

### Effects of modulated macrophages on osteogenic differentiation of HBMSCs

The effects of the macrophages modulated by CS ionic products (direct-modulated macrophages), or the macrophages modulated by conditioned medium of HBMSCs activated by CS ionic products (indirect-modulated macrophages) on the osteogenic differentiation of HBMSCs were further evaluated by using an indirect co-culture system illustrated in Fig.  1B. Briefly, THP-1 cells were differentiated to macrophages in a six-well plate at the density of 1 × 10^6^ cells per well and treated with 500 ng/ml LPS for 1 h before being incubated with RPMI CS 0, RPMI CS 1/64, RPMI CS 1/128, HBMSC CM 0, HBMSC CM 1/64 and HBMSC CM 1/128 for 48 h, respectively. Then, the conditioned medium of macrophages cultured with different RPMI media (RPMI CS 0, RPMI CS 1/64 and RPMI CS 1/128) and cultured with different HBMSC conditioned media (HBMSC CM 0, HBMSC CM 1/64, HBMSC CM 1/128) were collected, filtered and referred as CM CS 0, CM CS 1/64, CM CS 1/128 and CM CM 0, CM CM 1/64, CM CM 1/128, respectively. Meanwhile, HBMSCs were seeded in a 12-well plate at the density of 2.5 × 10^5^ per well and cultured with normal medium for 24 h, followed by incubation with osteogenic medium for further 48 h. Afterwards, the culture medium of HBMSCs were replaced with the collected conditioned media (CM CS 0, CM CS 1/64, CM CS 1/128 and CM CM 0, CM CM 1/64, CM CM 1/128) and the HBMSCs were continued to be cultured for another 4 days. The HBMSCs stimulated by conditioned medium of macrophages regulated by different CS ionic products and (RPMI CS 0, RPMI CS 1/64 and RPMI CS 1/128) and conditioned media of macrophages regulated by different CS-activated HBMSC conditioned media (HBMSC CM 0, HBMSC CM 1/64, HBMSC CM 1/128) were recorded as CS-Mφ-HBMSCs and CS-HBMSCs-Mφ-HBMSCs, respectively (Fig.  1B). The osteogenic differentiation of CS- Mφ-HBMSCs and CS-HBMSCs- Mφ-HBMSCs were then evaluated by Q-RT-PCR and alkaline phosphatase (ALP) staining assay, as described in section ‘Quantitative real-time polymerase chain reaction’ and following, respectively.

For ALP staining assay, after the HBMSCs were cultured with different conditioned medium for 4 d, the cells were fixed with 4% PFA for 10 min at room temperature prior to be washed three times with DPBS. Then, a BCIP/NBT Alkaline Phosphatase Color Development Kit (Beyotime, China) was used to detect the expression of ALP in HBMSCs according to the manufacturer’s instructions.

### Immunofluorescence staining assay

Immunofluorescence staining assay was applied to observe the expression and location of COX-2 in HBMSCs cultured with different media. Briefly, after HBMSCs were incubated with or without CS ionic products in confocal dishes for 24 h, the cells were washed two times with DPBS and fixed by 4% paraformaldehyde for 15 min. Then, the cells were permeabilized with 20% Triton for 5 min at room temperature and blocked with DPBS containing 1% BSA (Sigma) for 1 h at 37°C, followed by being incubated with primary antibody solution containing rabbit polyclonal anti-COX2 antibody (1:100, abcam, USA) for 2 h at 37°C and overnight at 4°C. Then, the cells were washed two times with DPBS and incubated with secondary antibody solution containing Alexa 488 goat anti-rabbit IgG antibody (1:1000, abcam) for 1 h at 37°C. Cell nuclei were revealed with 5 μg/ml 4,6-diamidino-2-phenylindole (DAPI) for 10 min at room temperature. Finally, the cells were observed with a laser confocal microscope (Leica SP5, Germany), and images were taken with a CCD camera (Leica DFC 420C). Three images were taken per sample, and the mean fluorescence intensity of each image was measured with ImageJ software.

### Western blot analysis

Western blot analysis was applied to determine the expression of COX-2 in HBMSCs. Briefly, after HBMSCs were incubated with or without CS ionic products for 24 h, the cells were washed twice with DPBS and lysed in RIPA lysis buffer containing 1% (v/v) phenylmethylsulfonyl fluoride at 4°C for 30 min. Then, the cell lysates were centrifuged at 10 000 g for 30 min, and the supernatant was collected. Protein concentrations in the supernatant were measured by using a Pierce^®^ BCA Protein Assay Kit (Thermo, USA) according to the manufacturer’s instructions. Next, 2× protein-loading buffer was added to 25 μg proteins, and the mixture was heated at 95°C for 5 min. Then, the proteins were separated by performing sodium dodecyl sulfate polyacrylamide gel electrophoresis with 10% gel, and then transferred to polyvinylidene difluoride membranes. The membranes were blocked with western blocking buffer (Beyotime, China) at room temperature for 1.5 h, and incubated with rabbit anti-COX2 antibody (1:500, abcam) and mouse anti-glyceraldehyde 3-phosphate dehydrogenase (GAPDH) antibody (1:500, abcam) overnight at 4°C, respectively. Afterwards, the membranes were washed three times with western washing buffer (Beyotime, China) and then incubated with horseradish peroxidase-conjugated anti-rabbit secondary antibody (1:1000, abcam) at room temperature for 2 h. At last, the immunoreactive protein bands were visualized by using a Tanon-5200 chemiluminescent imaging system (Tanon Science & Technology Co., Ltd, China) and quantified with ImageJ software.

### Quantitative real-time polymerase chain reaction

Gene expression of TSG 6, COX-2, PTGES2, hepatocyte growth factor (HGF), bone morphogenetic protein-2 (BMP-2), Collagen I (COL-1), ALP and Runt-related transcription factor 2 (RUNX2) in HBMSCs, and interleukin-1β (IL-1β), TNF-α, interleukin-10 (IL-10), transforming growth factor-β (TGF-β), CC-chemokine receptor 7 (CCR7) and CD163 in macrophages were detected by performing Q-RT-PCR. Briefly, total RNA of the cells was extracted using an E.Z.N.A. Total RNA Kit I (OMEGA, Biotek) followed by the manufacturer’s instructions. RNA concentration was measured using a nanodrop 1000 reader (Thermo Scientific), and a ReverTra Ace-α Kit (ToyoboCo. Ltd, Japan) was used to synthesize cDNA, according to the manufacturer’s instructions. Then, 0.1 μl cDNA was diluted with 4.1 μl sterilized deionized water, and 4.2 μl diluted cDNA was mixed with 5 μl SYBR-Green and 0.8 μl primers. The final concentration of primers in the mixture was 400 nM, and GAPDH was used as housekeeping gene. Q-RT-PCR analysis was performed on the mixture of cDNA and primers loaded in a 384-well plate using a 7900 Real-time PCR system (Applied Biosystems) for 40 cycles (95°C for 15 s, 60°C for 15 s, 72°C for 45 s) after the initial incubation step of denaturation at 95°C for 1 min. At last, 2^−^^ΔΔCt^ method was used to calculate the relative gene expression. The data were normalized to GAPDH and compared with the corresponding gene expression of the control group. All primers used in the study were purchased from Sangon Biotech Co. Ltd (Shanghai), and the primer sequences were listed in Table  1.

**Table 1.** Primer sequences used in this study

Gene	Forward primer (5′–3′)	Reverse primer (5′–3′)
GAPDH	GTATCGTGGAAGGACTCATGAC	ACCACCTTCTTGATGTCATCAT
IL-1β	GCCAGTGAAATGATGGCTTATT	AGGAGCACTTCATCTGTTTAGG
TNF-α	AGTCTGGGCAGGTCTACTTT	CGTTTGGGAAGGTTGGATGT
IL-10	CCAAGAGAAAGGCATCTACA	GGGGGTTGAGGTATCAGAG
TGF-β	ACAGCAACAATTCCTGGCGATACC	CTCAACCACTGCCGCACAACTC
CCR7	TGAGGTCACGGACGATTACAT	GTAGGCCCACGAAACAAATGAT
CD163	ATCAACCCTGCATCTTTAGACA	CTTGTTGTCACATGTGATCCAG
ALP	AGCCCTTCACTGCCATCCTGT	ATTCTCTCGTTCACCGCCCAC
RUNX2	CCAACCCACGAATGCACTATC	TAGTGAGTGGTGGCGGACATAC
BMP-2	AACACTGTGCGCAGCTTCC	CTCCGGGTTGTTTTCCCAC
COL-I	GAGGGCCAAGACGAAGACATC	CAGATCACGTCATCGCACAAC

### Statistical analysis

Data were presented as means ± standard deviation. At least three samples of each test were taken for statistical analysis. Statistical significance between the groups was assessed with Student’s *t*-test. Differences were considered significant when *P *<* *0.05 (*) or *P *<* *0.01 (**).

## Results

### Effects of CS ionic products on the expression of immunosuppressive factors in HBMSCs and the mechanism

**Figure 2.**
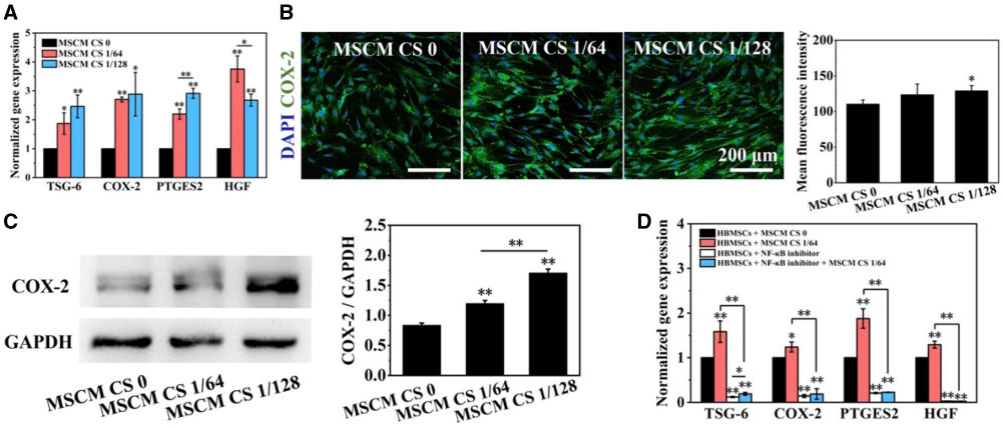
Effects of CS ionic products on the expression of immunosuppressive factors in HBMSCs. (**A**) Gene expression of COX-2, TSG-6, PTGES2 and HGF in HBMSCs cultured with CS ionic products (MSCM CS 1/64 and MSCM CS 1/128) or without CS ionic products (MSCM CS0). (**B**, **C**) Immunofluorescence staining and western blot analysis results of COX-2 in HBMSCs cultured with (MSCM CS 1/64 and MSCM CS 1/128) or without CS ionic products (MSCM CS0). (**D**) Effects of CS ionic products on the gene expression of immunosuppressive factors in HBMSCs pre-treated with or without NF-κB inhibitor. *P <* *0.05 and **P <* *0.01

After the HBMSCs were cultured with MSCM CS 0, MSCM CS 1/64 or MSCM CS 1/128 for 24 h, the gene expression of immunosuppressive factors (COX-2, TSG-6, PTGES2 and HGF) in HBMSCs were detected and the results are shown in Fig.  2A. Obviously, the addition of CS ionic products in the control medium (MSCM CS 1/64 or MSCM CS 1/128) significantly up-regulated these gene expression in HBMSCs when compared with the control medium (MSCM CS 0). Besides, the elevation of the gene expression of PTGES2 in HBMSCs cultured with MSCM CS 1/64 were higher than that in HBMSCs cultured with MSCM CS 1/128, while the elevation of the gene expression of HGF in HBMSCs cultured with MSCM CS 1/128 were higher than that in HBMSCs cultured with MSCM CS 1/64. In addition, although the gene expression results showed that the effects of MSCM CS 1/64 and MSCM CS 1/128 on the expression of TSG-6 and COX-2 in HBMSCs were similar, it was observed that the protein level of COX-2 in HBMSCs cultured with MSCM CS 1/128 was higher than that in HBMSCs cultured with MSCM CS 1/64 (Fig.  2B and C).

In addition, NF-κB inhibitors obviously reduced the expression of COX-2, TSG-6, PTGES2 and HGF in HBMSCs. Although MSCM CS 1/64 could significantly enhance the gene expression of COX-2, TSG-6, PTGES2 and HGF in HBMSCs, it did not exert stimulatory effects on the expression of immunosuppressive factors in the HBMSCs pre-treated with NF-κB inhibitors (Fig.  2D). These results suggest that the stimulatory effects of CS ionic products on the immunosuppressive factors in HBMSCs is dependent on the NF-κB pathway.

### Synergistical stimulatory effects of CS ionic products and pro-inflammatory cytokines on the immunomodulation function of HBMSCs

**Figure 3.**
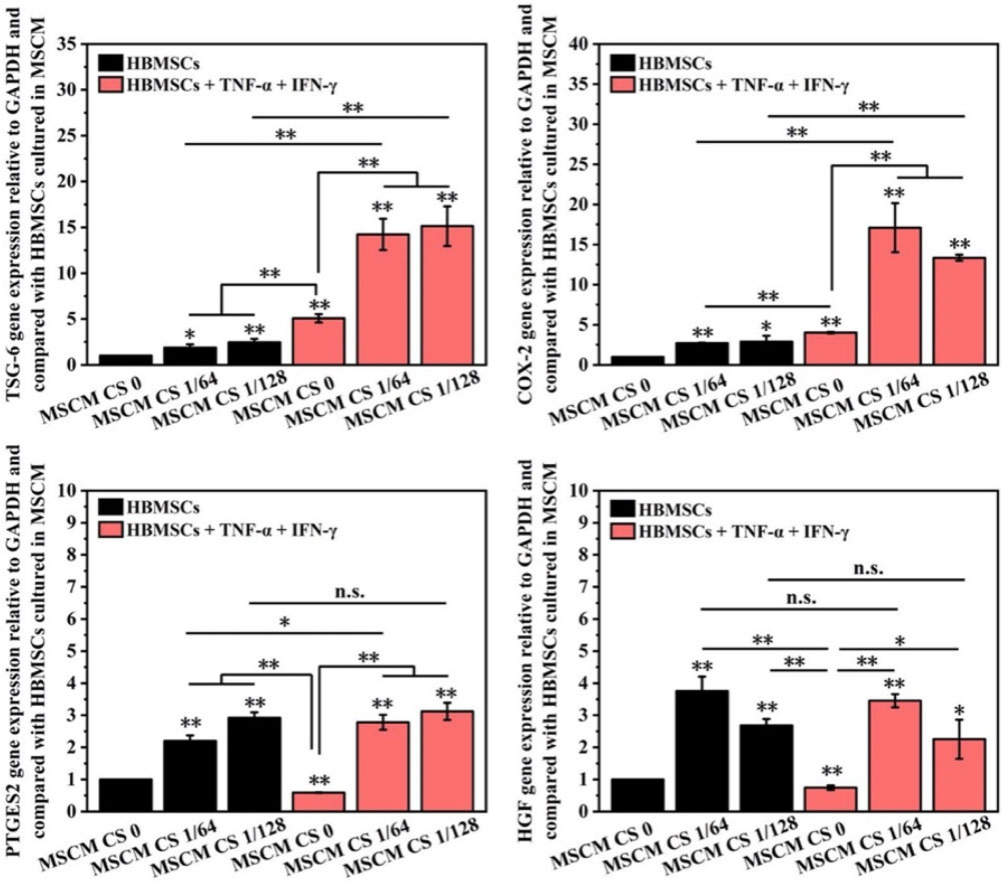
Effects of CS ionic products on the gene expression of immunosuppressive factors in HBMSCs treated with or without inflammatory factors. *P <* *0.05 and **P <* *0.01

Although CS ionic products in the culture medium can stimulate the gene expression of immunosuppressive factors (COX-2, TSG-6, PTGES2 and HGF) in HBMSCs, the treatment of pro-inflammatory factors IFN-γ + TNF-α showed stronger stimulatory effects on the expression of COX-2 and TSG-6 when compared with CS ionic products (Fig.  3). Specifically, the expression of TSG6 in HBMSCs cultured with MSCM CS 1/64 and MSCM CS 1/128 was 2 and 2.5 times higher than that in HBMSCs cultured with MSCM CS 0, respectively. The expression of TSG6 in HBMSCs cultured with IFN-γ + TNF-α was about five times higher than that in HBMSCs cultured with MSCM CS 0. Similar phenomena can be observed in the expression of COX-2. It was noting that, different from the stimulatory effects of CS ionic products on the gene expression of PTGES2 and HGF in HBMSCs, IFN-γ + TNF-α had no effects on enhancing the corresponding gene expression in HBMSCs.

Interestingly, it was found that CS ionic products could synergistically act with IFN-γ + TNF-α on enhancing the gene expression of COX-2 and TSG-6 in HBMSCs since the HBMSCs cultured with medium containing CS ionic products and IFN-γ + TNF-α had much higher gene expression of COX-2 and TSG-6 than those cultured with IFN-γ + TNF-α alone or CS ionic products alone. Specifically, the TSG-6 in HBMSCs cultured with medium containing CS ionic products and IFN-γ + TNF-α was about 14 times higher than that in HBMSCs cultured with MSCM CS 0. Similar trends can be observed in COX-2 expression in HBMSC cultured with different medium, and the COX-2 in HBMSCs cultured with medium containing MSCM CS 1/64 and IFN-γ + TNF-α was about 15 times higher than that in HBMSCs cultured with MSCM CS 0. However, there was no significant difference in the gene expression of PTGES2 and HGF between the CS groups and CS + IFN-γ + TNF-α groups, indicating no effects of IFN-γ + TNF-α on enhancing the corresponding gene expression in HBMSCs.

### Direct and indirect effects of CS ionic products on polarization of macrophages

**Figure 4.**
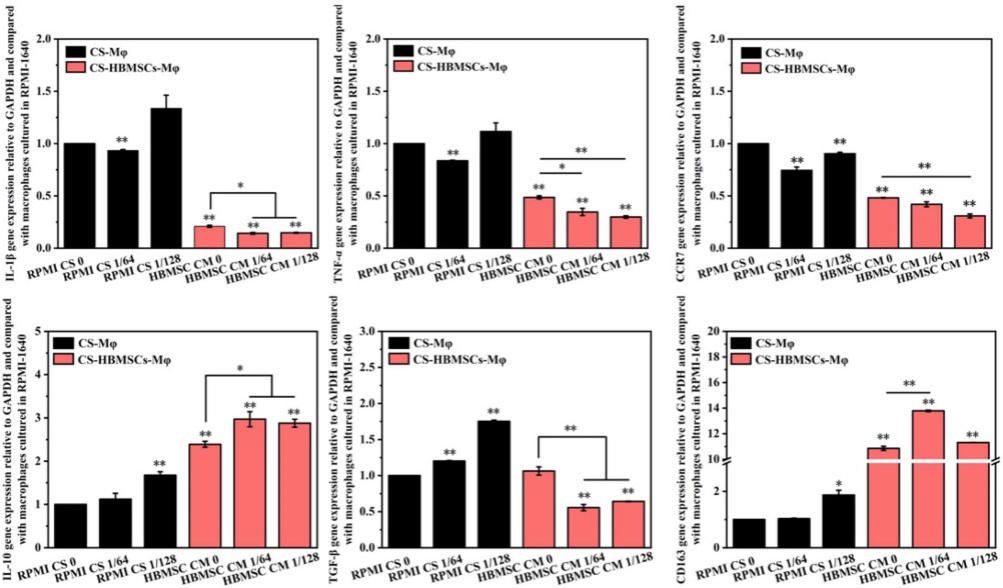
Gene expression of inflammation related factors (IL-1β, TNF-α, IL-10, TGF-β), M1 marker (CCR7) and M2 marker (CD163) of macrophages cultured with different medium. *P <* *0.05 and **P <* *0.01

To evaluate the direct and indirect effects of CS ionic products on polarization of macrophages, the effects of RPMI CS 0, RPMI CS 1/64 and RPMI CS 1/128 and conditioned medium from CS-activated HBMSCs (HBMSC CM 0, HBMSC CM 1/64, HBMSC CM 1/128) on the polarization of macrophages, which referred as CS-Mφ and CS-HBMSCs-Mφ, were investigated. It can be seen from Fig.  4 that the CS ionic products induced the polarization of macrophages toward M2 phenotype in some degree as the RPMI CS 1/64, and RPMI CS 1/128 down-regulated the gene expression of pro-inflammatory factors IL-1β, TNF-α and M1 marker CCR7 but up-regulated the gene expression of anti-inflammatory factor IL-10 and M2 marker CD163. More importantly, conditioned media from CS-activated HBMSCs showed stronger stimulatory effects on M2 polarization of macrophages than the CS ionic products. Specifically, gene expression of pro-inflammatory factors IL-1β, TNF-α and M1 marker CCR7 were significantly down-regulated while the gene expression of anti-inflammatory factor IL-10 and M2 marker CD163 were significantly up-regulated in macrophages cultured with conditioned medium from CS-activated HBMSCs (HBMSC CM 0, HBMSC CM 1/64, HBMSC CM 1/128) when compared with those in macrophages cultured with corresponding RPMI-1640 medium containing different concentrations of CS ionic products (RPMI CS 0, RPMI CS 1/64, and RPMI CS 1/128). In addition, when macrophages were cultured with conditioned medium of CS-activated HBMSCs, HBMSC CM 1/64 and HBMSC CM 1/128 showed stronger down-regulation effects on IL-1β, TNF-α and CCR7 genes and up-regulation effects on IL-10 when compared with HBMSC CM 0. Meanwhile, only HBMSC CM 1/64 but not HBMSC CM 1/128 showed stronger up-regulation effects on CD163 when compared with HBMSC CM 0. Moreover, significant down-regulation of fibrosis-enhancing factor TGF-β gene was also observed in macrophages cultured with HBMSC CM 1/64 or HBMSC CM 1/128 compared with that in macrophages cultured with HBMSC CM 0. Taken together, these results indicate that the immunosuppressive function of HBMSCs could be directly enhanced by CS ionic products (CS-Mφ), and the indirect effects of CS ionic products (CM of HBMSCs activated by CS ionic products) on regulating the polarization of macrophages from M1 to M2 were stronger than the direct effects.

**Figure 5.**
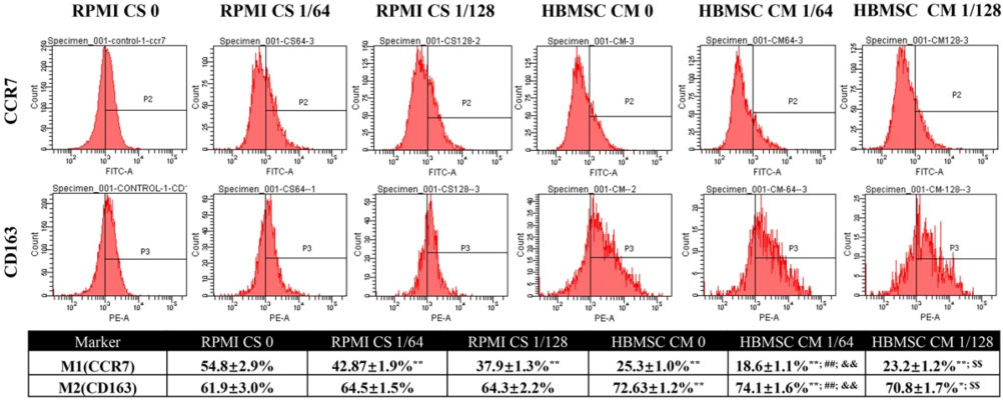
Flow cytometry results of macrophages cultured with different medium. *P <* *0.05 and **P <* *0.01 when compared with the data of RPMI CS 0. ^##^*P *<* *0.01 when compared with the data of HBMSC CM 0. ^&&^*P *<* *0.01 when compared with the data of RPMI CS 1/64. ^$$^*P *<* *0.01 when compared with the data of RPMI CS 1/128

The polarization of macrophages was further evaluated by evaluating the expression of M1 marker CCR7 and M2 marker CD163 in the cells using flow cytometry and the results are shown in Fig.  5. It can be seen that the conditioned media from CS-activated HBMSCs showed stronger stimulatory effects on M2 polarization of macrophages than the CS ionic products as the proportion of macrophages expressing M2 marker CD163 in macrophages cultured with conditioned medium from CS-activated HBMSCs (HBMSC CM 0, HBMSC CM 1/64, HBMSC CM 1/128) was significantly higher when compared with those in macrophages cultured with corresponding RPMI-1640 medium containing different CS ionic products (RPMI CS 0, RPMI CS 1/64 and RPMI CS 1/128). Meanwhile, the proportion of macrophages expressing M1 marker CCR7 cultured with the conditioned media from CS-activated HBMSCs (HBMSC CM 0, HBMSC CM 1/64, HBMSC CM 1/128) was significantly lower than those cultured with corresponding RPMI-1640 medium containing different CS ionic products (RPMI CS 0, RPMI CS 1/64 and RPMI CS 1/128), while the proportion of macrophages expressing M2 marker CD163 cultured with the conditioned media from CS-activated HBMSCs (HBMSC CM 0, HBMSC CM 1/64 and HBMSC CM 1/128) was significantly higher than those cultured with corresponding RPMI-1640 medium containing same CS ionic products (RPMI CS 0, RPMI CS 1/64 and RPMI CS 1/128). In addition, when the macrophages were cultured with conditioned media from CS-activated HBMSCs, only HBMSC CM 1/64 but not HBMSC CM 1/128 showed stronger regulatory effects on polarization of macrophages when compared with the HBMSC CM 0 group. In addition, both HBMSC CM 1/64 and HBMSC CM 1/128 had no effects on increasing the proportion of macrophages expressing M2 marker CD163.

### Effects of modulated macrophages on osteogenic differentiation of HBMSCs

**Figure 6.**
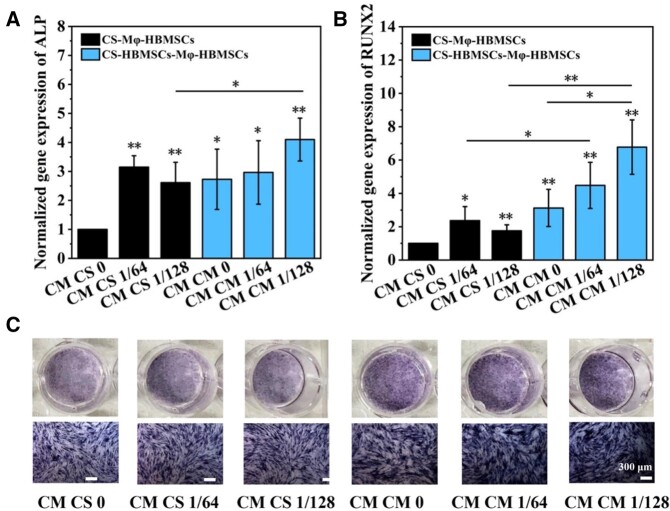
(**A**) ALP Expression in HBMSCs cultured with different medium. (**B**) RUNX2 expression in HBMSCs cultured with different media. (**C**) ALP staining analysis of HBMSCs cultured with different medium. *P <* *0.05 and **P <* *0.01

The effects of macrophages activated by CS ionic products (CS-Mφ) and conditioned medium from CS-activated HBMSCs (CS-HBMSCs-Mφ) on *in vitro* osteogenic differentiation of HBMSCs were investigated and the results are shown in Fig.  6. Figure  6A and B shows that the conditioned medium collected from macrophages regulated by CS ionic products (CM CS 1/64 and CM CS 1/128) enhanced the gene expression of early osteogenic differentiation markers of ALP and RUNX2 in HBMSCs when compared with the conditioned medium derived from macrophages cultured with RPMI-1640 (CM CS 0). However, there were no significant difference between the CM CS 1/64 and CM CS 1/128 groups. Similarly, the conditioned medium collected from macrophages stimulated by conditioned medium of CS-activated HBMSCs (CM CM 1/64 and CM CM 1/128) enhanced the gene expression of early osteogenic differentiation markers of ALP and RUNX2 in HBMSCs when compared with the conditioned medium collected from macrophages stimulated with conditioned medium of HBMSC cultured with control medium cultured (CM CM 0). In addition, the CM CS 1/128 medium showed stronger stimulatory effects on ALP and RUNX 2 gene expression than the CM CS 1/64. Interestingly, all conditioned media collected from macrophages stimulated by CS-activated HBMSCs showed much stronger stimulatory effects on the RUNX2 expression in HBMSCs when compared with all conditioned media collected from macrophages regulated by CS ionic products, which indicated that the conditioned medium collected from macrophages stimulated by CS-activated HBMSCs might have stronger stimulatory effects than the conditioned medium collected from macrophages regulated by CS ionic products. The expression of ALP was further confirmed by ALP staining assay, and the results were in accordance with the gene expression results (Fig.  6C).

## Discussion

In recent years, more and more evidence has shown that the coordinated interaction of macrophages and MSCs plays a critical role in the process of successful bone regeneration. As the importance of inflammatory responses in the process of bone regeneration has been widely valued, many studies in recent years have confirmed that BSCs can modulate the inflammation microenvironment by affecting the macrophage behaviors to promote the osteogenic differentiation of MSCs [17, 18, 20, 42]. However, whether the BSCs can influence the immunomodulatory function of MSCs and subsequently indirectly affect the macrophage behaviors to regulate the inflammation microenvironment for promoting osteogenic differentiation of MSCs are rarely studied. In this study, we proved that the CS ionic products could significantly enhance the immunoregulatory function of HBMSCs via NF-κB pathway. Furthermore, these CS-activated HBMSCs could stimulate the macrophages to polarize toward M2 phenotype, which created a favorable immune microenvironment to enhance the osteogenic differentiation of HBMSCs.

There are several studies showing that BSCs have the abilities of reducing the expression of inflammatory factors and promoting the polarization of macrophages to the M2 phenotype [17, 18, 20, 42], and the same results were obtained in the current study, indicating that the regulation effects of BSCs on the phenotypic polarization of macrophages may be one of the key reasons for the material to regulate the inflammatory response. More and more studies have demonstrated that successful bone healing is based on carefully coordinated crosstalk between inflammatory and bone forming cells [16, 17, 22, 23, 43, 44]. During this process, M2 macrophages play key roles in the recruitment and regulation of the differentiation of MSCs during bone regeneration and animal studies have comprehensively demonstrated that bone fractures do not heal without the direct involvement of macrophages [16, 17, 23, 45]. In these studies, MSCs have been recognized as stem cells that have osteogenic differentiation ability to participate the bone forming, i.e. MSCs have been allocated as bone forming cells.

When the BSCs are applied for bone regeneration, many studies have confirmed that BSCs participate the crosstalk between immune cells and bone forming cells by modulating the behaviors of macrophages as well as the inflammation microenvironment of bone defect, which subsequently enhance the bone healing and regeneration [16]. Most of the interactions between material, macrophage and MSCs reported in these literatures focused on the BSC modulation of macrophages to create a regulated inflammation microenvironment to act on MSCs. However, emerging evidence has shown that MSCs not only have the osteogenic differentiation ability but also have the immunomodulation ability [29, 46]. MSCs are known to deploy an anti-inflammatory effect and polarize M1 macrophages into M2 macrophages, thus modulating inflammation and launching bone repair [47]. In this study, the immunomodulatory ability of BSC on MSCs was considered, endowing the MSCs with two roles, i.e. immune cells and stem cells. We firstly reported that the immunosuppressive function of MSCs was enhanced by CS ionic products and the CS-activated HBMSCs had even a stronger promotion effect on M2 polarization of macrophages than CS ionic products. Studies have reported that M2 macrophages with anti-inflammatory ability have significantly more potential to strengthen bone regeneration compared with naive (M0) and classically activated macrophages (M1) [48–50]. Thus, the indirect immune regulation of CS may contribute more on bone regeneration than the direct regulation. Taken together, BSCs can not only directly regulate the polarization of macrophages by their ionic products but also indirectly modulate the polarization of macrophages through affecting the immunosuppressive abilities of MSCs. More importantly, the immunomodulatory efficiency of BSC activated-MSCs is higher than the BSC ionic products.

It is well known that the four immunosuppressive factors (TSG-6, COX-2, PTGES2 and HGF) of MSCs play important roles in modulating inflammation responses [27, 28, 51, 52]. For example, TSG-6 and PGE2 are two key molecules for MSCs to regulate the polarization of macrophages to the M2 phenotype. It has been reported that TSG-6 can down-regulate the activity of the NF-κB signaling pathway in macrophages and inhibit the expression of TNF-α and other pro-inflammatory factors, thereby weakening the pro-inflammatory cascade mediated by macrophages [27] while PEG2 can bind to the EP2 and EP4 receptors on the surface of macrophages to promote the conversion of macrophages from M1 phenotype to M2 phenotype, and then produce a large amount of anti-inflammatory factor IL-10 to inhibit inflammation [28]. It has well known that MSCs can exert their immunomodulatory properties by directly contacting with immune cells or/and by secreting regulatory molecules [47]. In this study, the expression of the four immunosuppressive factors of HBMSCs was significantly enhanced by CS ionic products, which might contribute to the strong stimulatory effects of conditioned media of CS-activated HBMSCs on M2 polarization of macrophages. In addition, these up-regulated immunosuppressive factors may have higher working efficiency than CS ionic products since they can directly interact with macrophages. CS ionic products mainly contain calcium ions and silicate ions. The important role of silicate ions in the effects of BSCs on behaviors of different types of cells have been widely reported in our previous studies [12, 13]. In these previous studies, we found that, when the CS ionic products were diluted with cell culture medium at ratios of 1/64 and 1/128, the final ion concentration of calcium ions in the diluted media was usually similar to that in the original cell culture medium due to the existence of large amount of calcium ions in the original cell culture medium. However, the final ion concentration of silicate ions in the diluted media was significantly higher as there were almost no silicate ions in the original cell culture medium. Thus, it is mainly the silicate ions in the CS ionic products that contribute to the regulatory effects of CS ionic products on cell behaviors. In this study, the CS ionic products containing the silicate ions at effective concentrations (1.8 µg/ml for 1/64 and 1.25 µg/ml for 1/128 dilution) reported in our previous study were chosen to directly treat HBMSCs and the results confirmed that the CS ionic products were effective for regulating the immunomodulatory function of HBMSCs. The silicate ions at these two concentrations have also been reported to be able to simulate the osteogenic differentiation of MSCs and vascularization of endothelial cells, which are important for the proliferation stage of bone regeneration. These results further indicate that a BSC or a composite material containing BSC that can release the silicate ions at these effective concentrations may be able to regulate the whole bone regeneration process, including the inflammation, proliferation and tissue remodeling stages.

**Figure 7.**
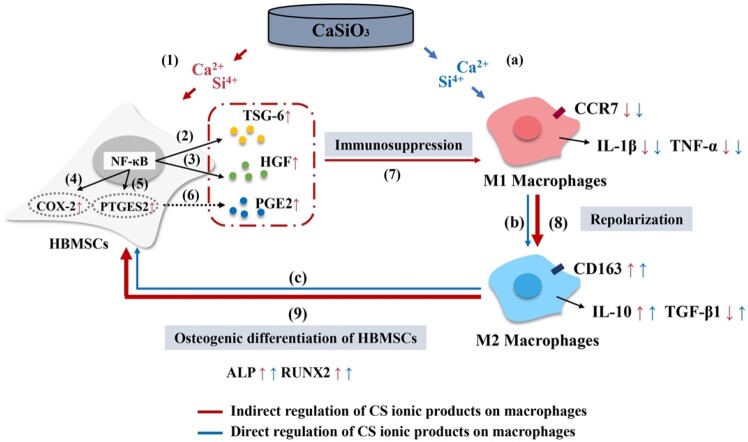
The proposed mechanism of CS ionic products promoting *in vitro* osteogenic differentiation of HBMSCs through directly modulating the behaviors of macrophages or indirectly modulating the behaviors via regulating the immunosuppressive function of HBMSCs

The proposed mechanism of which CS ionic products stimulating osteogenic differentiation is shown in Fig.  7, which indicates that CS ionic products can promote *in vitro* osteogenic differentiation of HBMSCs through directly modulating the macrophages (blue line, Steps a–c) or indirectly stimulating the macrophages (red line, Steps 1–9) to polarize into M2 phenotype by enhancing immunosuppressive function of HBMSCs. This illustration shows that the crosstalk between immune cells and bone forming cells advances from a straight line (BSC–macrophage–MSCs) into a loop (BSC–MSCs–macrophage–MSCs) after the immunomodulation ability of MSCs are considered in this study. Since the current study can mimic the real microenvironment of the bone defects where MSCs participate to the inflammation regulation, the mechanism of BSC enhancing bone regeneration has been further elucidated through this study. More importantly, the current study suggests that the effects of biomaterials on immunomodulatory function of tissue forming cells involved in wound healing are also critical and should be considered in future studies on the immunomodulatory function of biomaterials.

## Conclusion

In this study, for the first time, we found that the CS ionic products could significantly enhance the immunosuppressive function of HBMSCs via NF-κB pathway. In addition, these CS-activated HBMSCs showed stronger stimulatory effects on M2 phenotype polarization of macrophages than CS ionic products, which indicates that the CS ions, especially the silicate ions, released from CS can regulate the inflammation microenvironment by indirectly modulating the polarization of macrophages via regulating the immunomodulatory function of MSCs. Furthermore, the inflammation microenvironment created by the macrophages regulated by CS-activated HBMSCs showed higher stimulatory effects on osteogenic differentiation of HBMSCs than the one created by macrophages modulated by CS ionic products. Thus, the regulatory effects of CS ionic products, especially the silicate ions, on the immunomodulatory function of MSCs are also critical for creating a favorable inflammation microenvironment for bone regeneration. These results provide further understanding on the interactions between BSCs, MSCs and macrophages by considering the immunomodulation ability of MSCs, which further contribute to the studies on the mechanism by which CS stimulates osteogenic differentiation and enhances bone regeneration. In addition, these results suggest that it is more important to consider the effects of biomaterials on the immunomodulatory function of the tissue forming cells involved in tissue regeneration process than to investigate the effects of biomaterials on immune cells when the immunomodulatory function of biomaterials is investigated.

## Funding

This work was supported by the National Natural Science Foundation of China (Grant Nos. 31771024 and 31971274).

*Conflict of interest statement.* None declared.
